# Memory decay and susceptibility to amnesia dissociate punishment- from relief-learning

**DOI:** 10.1098/rsbl.2012.1171

**Published:** 2013-08-23

**Authors:** Sören Diegelmann, Stephan Preuschoff, Mirjam Appel, Thomas Niewalda, Bertram Gerber, Ayse Yarali

**Affiliations:** 1 Department of Genetics of Learning and Memory, Leibniz Institute for Neurobiology (LIN), Magdeburg, Germany; 2 Research Group Molecular Systems Biology of Learning, Leibniz Institute for Neurobiology (LIN), Magdeburg, Germany; 3 Max-Planck Institute of Neurobiology, Martinsried, Germany; 4 Center for Behavioral Brain Sciences, Magdeburg, Germany

**Keywords:** *Drosophila melanogaster*, memory stability, punishment-learning, relief-learning, retrograde amnesia, reinforcement

## Abstract

Painful events shape future behaviour in two ways: stimuli associated with pain onset subsequently support learned avoidance (i.e. punishment-learning) because they signal future, upcoming pain. Stimuli associated with pain offset in turn signal relief and later on support learned approach (i.e. relief-learning). The relative strengths of such punishment- and relief-learning can be crucial for the adaptive organization of behaviour in the aftermath of painful events. Using *Drosophila*, we compare punishment- and relief-memories in terms of their temporal decay and sensitivity to retrograde amnesia. During the first 75 min following training, relief-memory is stable, whereas punishment-memory decays to half of the initial score. By 24 h after training, however, relief-memory is lost, whereas a third of punishment-memory scores still remain. In accordance with such rapid temporal decay from 75 min on, retrograde amnesia erases relief-memory but leaves a half of punishment-memory scores intact. These findings suggest differential mechanistic bases for punishment- and relief-memory, thus offering possibilities for separately interfering with either of them.

## 1. Introduction

A painful event has two sides: A ‘negative’ aspect at its onset and a ‘positive’ aspect at its offset, at the moment of relief [1]. Flies, for example, avoid an odour once it has been associated with the onset of an electric shock (odour → shock; henceforth called punishment-learning); yet they approach an odour once it has been associated with shock offset (shock → odour; henceforth called relief-learning; [2,3]). Similar Janus-headed results are found in rats and man: visual cues associated with shock onset potentiate startle, whereas cues associated with shock offset attenuate startle [4,5]. Thus, if embedded into a natural string of events, the net effect of such an adverse life event may depend on the relative strengths of oppositely valenced memories related to its onset (punishment-memory) and its offset (relief-memory). Here, we compare the time course of decay, as well as the susceptibility to post-training cold-amnesia, between these two kinds of memory. The differences we find in both these parameters suggest a dissociation of the mechanisms underlying punishment- and relief-memories.

## 2. Material and methods

Training and testing followed standard methods with the modifications described in detail in Yarali *et al.* [3] and electronic supplementary material, figure S1. Please note that these modifications, necessary to reveal relief-memory, make the procedure about sixfold more laborious than the standard protocol: a cohort of 100–150 wild-type Canton-S flies was differentially trained such that odour *X* was paired with electric shock, while odour *Y* was not. Then, the flies were offered the choice between both odours in a T-maze and the flies in either arm of the maze were counted. A preference index (Pref) was calculated as (all Pref values are documented in the electronic supplementary material, figures S2 and S3):
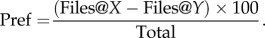
The assignment of benzaldehyde (BA) and 3-octanol (OCT) as *X* and *Y* was balanced across experiments, which allowed calculation of an associative learning index (LI) as:
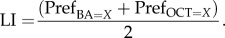
Negative LIs therefore indicate conditioned avoidance, whereas positive LIs indicate conditioned approach. Importantly, for punishment-training, odour *X* was presented before shock onset, whereas for relief-training it was presented upon shock offset; six training trials were concatenated in both cases (for details, see the electronic supplementary material, figure S1).

### (a) Memory decay

**Figure 1.**
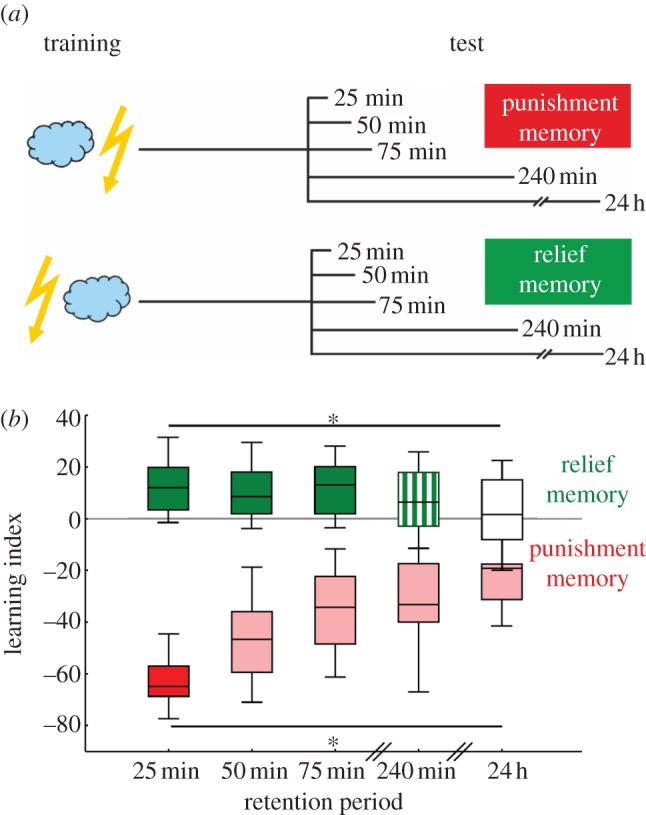
Different decay of punishment- and relief-memory. (*a*) Schematic of experimental design. (*b*) Punishment- and relief-memory assayed after retention periods of either 25, 50, 75, 240 min, or 24 h. Light shading indicates a difference from the scores at the earliest retention period (25 min), lack of fill indicates lack of significance from chance (i.e. from zero). For the 240 min retention period, relief-memory scores were neither different from the scores obtained at 25 min, nor from zero (hatched fill). Box-whisker plots (see §2 for details) show learning indices, positive values indicating conditioned approach to the trained odour (i.e. relief-memory) and negative values conditioned avoidance (i.e. punishment-memory). *p < 0.05 in KW-tests.

This experiment used a 2 × 5 experimental design (figure 1*a*), such that flies underwent either punishment- or relief-training, and were then tested after retention periods of 25, 50, 75,240 min, or 24 h. During the retention period and in the intervals between training trials, flies were kept in their regular food vials. These vials either remained within the experimental room yet outside the conditioning apparatus (50, 75 and 240 min retention periods) or were transferred overnight to the culture facility (24 h retention period).

### (b) Resistance to cold-amnesia

**Figure 2.**
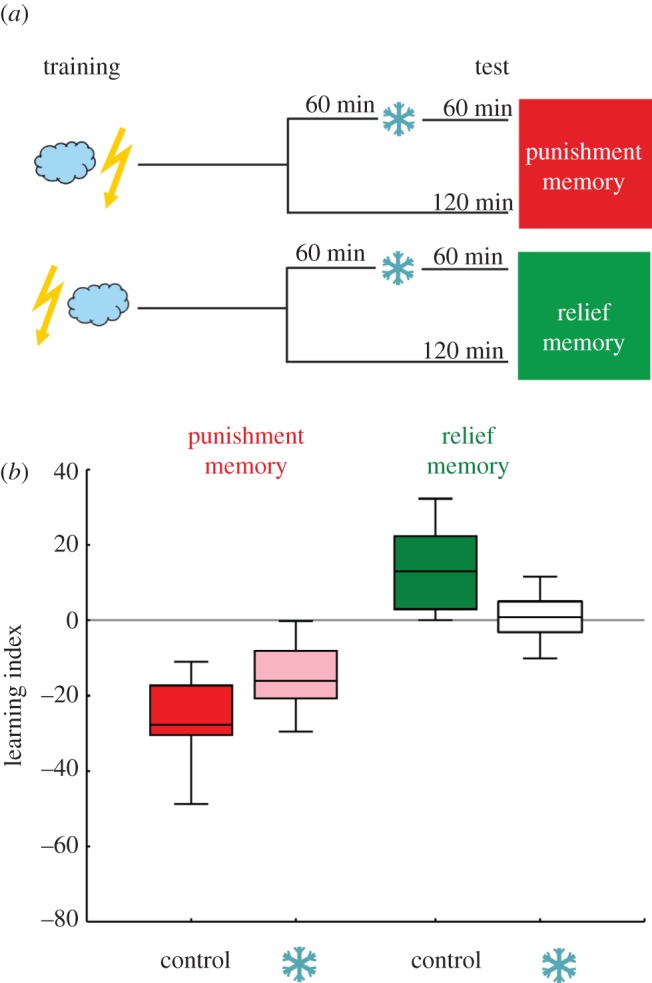
Punishment- and relief-memory differ in sensitivity for cold-amnesia. (*a*) Schematic of experimental design. (*b*) Punishment- and relief-memory assayed after 120 min retention period. For each kind of memory, one group underwent cold-amnesia treatment 60 min after training, while the other did not (i.e. control). Differences in shading indicate *p* < 0.05 in the respective MWU test, and lack of shading indicates lack of significance from chance performance (i.e. from zero).

This experiment used a 2 × 2 experimental design (figure 2*a*), such that flies underwent either punishment- or relief-training, and then either did or did not receive cold-amnesia at 60 min after training. Testing then took place 60 min later, i.e. at 120 min after training. Cold-amnesia was implemented by transferring flies into ice-cold plastic vials and then keeping them on ice for 2 min before transferring them back to regular food vials.

### (c) Statistics

Non-parametric statistics were used throughout. Kruskal–Wallis (KW) tests and Mann–Whitney *U* (MWU) tests were used to compare LI values between multiple and two groups of flies, respectively. One-sample sign tests (OSS) were used to determine whether scores of a given group were significantly different from zero. Throughout, a significance level of *p* < 0.05 was adopted. For multiple comparisons within a dataset, critical *p*-levels were adjusted by a Bonferroni correction (*p* < 0.05 divided by the number of comparisons) to maintain the experiment-wide error rate at 5 per cent. Data are plotted as box plots, representing the median as the middle line, the 25 and 75 per cent quantiles as boundaries of the box and the 10 and 90 per cent quantiles as whiskers.

## 3. Results

### (a) Memory decay

In terms of absolute learning scores (figure 1*b*), punishment-memory is obviously much stronger than relief-memory, as has been reported previously [2,3]. For the current context, it is important that punishment-memory scores decay as the retention period lengthens (KW: *p* < 0.05, *N* = 20 in all cases, d.f. = 4, *H* = 36.69; MWU comparing later retention periods to the earliest one (25 min): *p* < 0.05/4 in all cases, *U* = 97.00, 57.00, 62.00, 12.00). In fact, within 75 min punishment-memory has decayed to approximately 50 per cent of the initial score. Critically, however, punishment-memory remains detectable for at least 24 h (OSS for each retention period: *p* < 0.05/5 in all cases).

Relief-memory scores also change with lengthening of the retention period (KW: *p* < 0.05; *N* = 51, 35, 46, 43, 40; d.f. = 4, *H* = 13.82), but in a different way from punishment-memory scores. That is, compared with the shortest retention interval of 25 min, relief-memory remains stable during at least the first 240 min (MWU: *p* > 0.05/4 in all cases, *U* = 839.00, 1161.00, 833.00; mind the tendencial difference for the 240 min retention period). At 24 h after training, however, relief-memory scores have decayed significantly (MWU: *p* < 0.05/4; *U* = 634.5). Fittingly, relief-learning memory scores are significantly positive only for retention periods of up to 75 min (OSS: *p* < 0.05/5 in all cases except for 240 min, where *p* = 0.014 and 24 h, where *p* = 0.4).

### (b) Resistance to cold-amnesia

Despite decaying more slowly than punishment-memory within the first 240 min after training (figure 1), relief-memory seems more sensitive to retrograde amnesia. Implementing cold-amnesia 60 min after training eliminates the relief-memory scores measured another 60 min later, i.e. after a 120 min retention period (figure 2; MWU comparing the cold-anaesthesia group to control: *p* < 0.05, *U* = 41.00, *N* = 14, 14; OSS: *p* < 0.05/2 for the control group but *p* = 0.79 for the cold-amnesia group). The same treatment reduces punishment-memory scores only to about half (MWU: *p* < 0.05, *U* = 54.00, *N* = 14, 14; OSS: *p* < 0.05/2 for each group).

## 4. Discussion

We provide the first systematic comparison of the temporal dynamics of punishment- versus relief-memory. Over the first 4 h following training, relief-memory decays much slower than punishment-memory (figure 1*b*). This slow decay is reminiscent of the slow initial decay rate of sugar reward-memory [6–8]. With respect to longer retention periods, however, relief-memory differs from both punishment- and reward-memories: multiple, temporally spaced training trials result in detectable punishment-memory beyond 24 h [9,10], whereas for such long-term reward-memory, a single training trial suffices [8,11]. For relief-learning, despite using multiple, spaced training trials, we find no appreciable memory scores at 24 h (figure 1*b*). Thus, the temporal pattern of decay for relief-memories differs from both punishment- and reward-memories.

Regarding cold-amnesia, both punishment- and reward-memories are only partially susceptible within the first 2 h following training [6–10,12–14]. That is, cold-amnesia typically spares a so-called amnesia-resistant component of reward- and of punishment-memory. Indeed, we confirm that punishment-memory 1 h after training is composed of an amnesia-sensitive component and an amnesia-resistant component (figure 2). Critically, however, cold-amnesia abolishes relief-memory completely (figure 2). Given that for punishment-memory, anaesthesia-sensitive versus -resistant components of memory seem to have partially different genetic requirements (for proper function of e.g. the *amnesiac* and *radish* [12], *rutabaga* [10], *synapsin* [13] and *bruchpilot* [14] genes), it would be interesting to look for roles of these genes in relief-learning.

If, within a single subject, the events before and after a traumatic episode were to induce punishment- and relief-memory, our finding that both these forms of memory differ in strength and susceptibility to retrograde amnesia may be of practical importance: while trying to erase punishment-memory, one may unwittingly also erase relief-memory. Dependent on the relative strength of these memories and the relative effectiveness of the treatment, the net effect of such manipulation may make the overall-mnemonic effect of the traumatic episode even more adverse.
